# Highly Responsive Robotic Prosthetic Hand Control Considering Electrodynamic Delay

**DOI:** 10.3390/s25010113

**Published:** 2024-12-27

**Authors:** Jiwoong Won, Masami Iwase

**Affiliations:** Department of Robotics and Mechatronics, Tokyo Denki University, Tokyo 120-8551, Japan; won19@ctrl.fr.dendai.ac.jp

**Keywords:** user’s intention, electromyography (EMG), electro-mechanical delay (EMD), NARX model, zero-phase error tracking control (ZPETC)

## Abstract

As robots become increasingly integrated into human society, the importance of human–machine interfaces continues to grow. This study proposes a faster and more accurate control system for myoelectric prostheses by considering the Electromechanical Delay (EMD), a key characteristic of Electromyography (EMG) signals. Previous studies have focused on systems designed for wrist movements without attempting implementation. To overcome this, we expanded the system’s capability to handle more complex movements, such as those of fingers, by replacing the existing four-channel wired EMG sensor with an eight-channel wireless EMG sensor. This replacement improved the number of channels and user convenience. Additionally, we analyzed the communication delay introduced by this change and validated the feasibility of utilizing EMD. Furthermore, to address the limitations of the SISO-NARX model, we proposed a MISO-NARX model. To resolve issues related to model complexity and reduced accuracy due to the increased number of EMG channels, we introduced ridge regression, improving the system identification accuracy. Finally, we applied the ZPETC+PID controller to an actual servo motor and verified its performance. The results showed that the system reached the target value approximately 0.240 s faster than the response time of 0.428 s without the controller. This study significantly enhances the responsiveness and accuracy of myoelectric prostheses and is expected to contribute to the development of practical devices in the future.

## 1. Introduction

The 21st century can be considered an era of mechatronics, including robotics, as robots are increasingly integrated into human society. Among these advancements, myoelectric prostheses have emerged as transformative devices that replace parts of the human body. Unlike body-powered prostheses, myoelectric prostheses leverage EMG signals as control inputs, enabling a range of natural movements, such as hand opening and closing. According to Ottobock, these prostheses provide strong grip force with minimal effort and offer flexible hand movements regardless of arm posture, significantly enhancing the user’s ability to perform everyday tasks in various environments [1,2,3].

Research on myoelectric prostheses has primarily focused on improving hand gesture recognition and movement prediction using machine learning techniques such as neural networks (NNs), convolutional neural networks (CNNs), and multi-scale convolutional neural networks (MSCNNs) [4,5]. While these methods show high accuracy in recognizing hand patterns, achieving real-time performance and responsiveness remains a challenge, particularly as the complexity of movements and the amount of EMG data increase.

One critical challenge in human–machine interfaces, including myoelectric prostheses, is the delay between user intent and the system’s response. Delays exceeding 300 ms have been shown to negatively impact user performance in remote operations [6]. To address this issue, researchers have explored Electro-Mechanical Delay (EMD), which refers to the time lag (typically 30–100 ms) between the generation of EMG signals and the onset of movement. By leveraging EMD, myoelectric systems can predict user intent in advance, thereby reducing the overall response time [7]. Recent studies, such as Tigrini et al. (2023), have demonstrated the effectiveness of transient EMG data in predicting movement intent within a 150 ms window centered on movement onset, highlighting the applicability of EMD in anticipatory control systems [8].

Hayashi et al. also proposed utilizing EMD to improve the responsiveness of the system. Additionally, they proposed a Single Input–Single Output (SISO)-NARX model for wrist angle estimation to predict wrist movements and control a single-link manipulator. In their approach, the input signal was synthesized by combining the EMG signals obtained from a four-channel EMG sensor into a single signal. To enhance the manipulator’s responsiveness and accuracy, a controller combining feedforward and feedback control, specifically a ZPETC+PID controller, was employed. Hayashi et al. set the practical operation of devices such as myoelectric prostheses as a future research goal [7].

Based on the foundational research by Hayashi et al., this study aims to address the challenges encountered during practical implementation. Hayashi et al.’s approach combined four EMG signals into a single input for a SISO-NARX model, which failed to fully utilize the unique characteristics of each EMG channel. This inherently limited the system’s scalability, particularly in handling complex multi-degrees-of-freedom (Multi-DOF) movements, such as finger articulation. Additionally, Hayashi et al. utilized a four-channel wired sensor to acquire EMG signals, which required precise identification of muscle positions and adjustments during attachment. This process could cause inconvenience for users during operation and adjustment, potentially reducing user comfort and usability [9]. However, utilizing a band-type wireless sensor offers the advantage of providing a simple interface that users can easily wear, enabling signal acquisition without the need for complex equipment [10,11].

To address the scalability issues related to recognizing and estimating finger movements in addition to wrist motions, we propose replacing the four-channel wired sensor with an eight-channel band-type wireless sensor. This approach allows for acquiring EMG signals from eight channels instead of the original four channels, thereby enabling the system to estimate complex movements, such as finger motions, and ensuring scalability. However, switching from a four-channel wired sensor to an eight-channel wireless sensor is expected to introduce communication delays. While Hayashi et al.’s research considered only the motor dynamics using wired sensors, switching to a wireless sensor is expected to introduce communication delay issues. We will verify this communication delay and investigate the applicability of Electro-Mechanical Delay (EMD) in such conditions. As the angle estimation model, we propose using a MISO-NARX model with ridge regression [12], which is expected to prevent overfitting and improve generalization performance. Although it is generally known in the research domain of the system identification that multi-input models tend to have reduced identification accuracy due to increased complexity compared to the SISO-NARX model, we anticipate that ridge regression will enhance the performance of the MISO-NARX model, making it superior to the SISO-NARX model. The performance of ZPETC+PID control has been validated in prior studies. However, there are differences in the control targets between previous studies and this research. Therefore, we will verify whether the same control method applied to the servo motor, which is the control target in this study, produces results consistent with prior findings and confirm that there are no issues during practical implementation.

A systematic literature review was conducted using major academic databases, including IEEE Xplore, PubMed, and Google Scholar, with keywords such as “MISO NARX model”, “ridge regression EMG control”, and “multi-channel EMG analysis”. Despite extensive searches within the past five years, no studies were found that expanded upon the work of Hayashi et al. to integrate a MISO-NARX model or an eight-channel EMG configuration. This absence of prior work highlights the novelty of this research, which pioneers the application of advanced multi-channel configurations in wrist angle estimation systems.

By developing a scalable and responsive interface, this study paves the way for myoelectric prostheses that can perform complex, user-defined motions. In the future, this approach could enable prosthetic devices to execute versatile and intuitive movements, transforming them from tools of functional replacement to systems that mimic natural human motion.

## 2. Materials and Methods

### 2.1. Latency Assessment for Robotic Hand Motion

**Figure 1:**
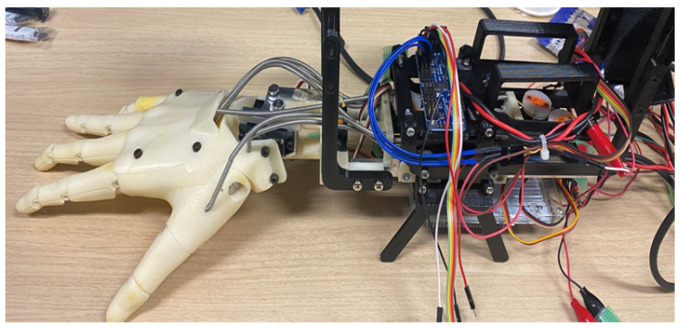
Robot hand.

**Figure 2:**
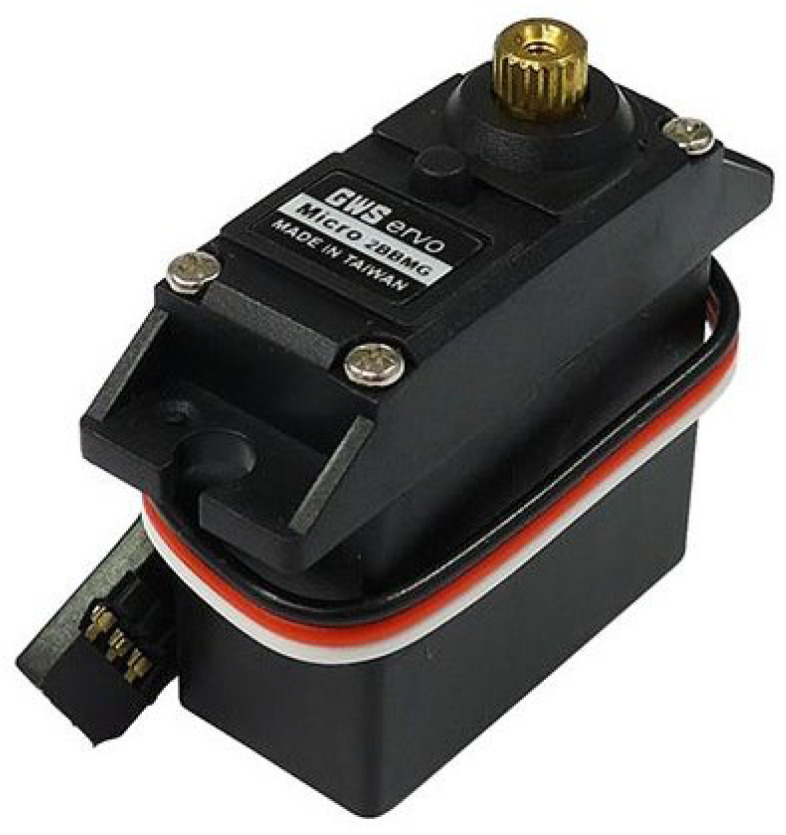
GWS Micro 2BBMG—micro servo manufactured by Grand Wing Servo-Tech Co., Ltd. (GWS), a company based in Taipei, Taiwan.

To conduct research on myoelectric prostheses, we utilized a hand-shaped robotic system, as shown in Figure 1. This robot features five motors for finger movement and two motors for wrist movement, simulating the joints of a hand. As an initial step toward implementing full hand movements, we are currently focusing on wrist movements only. The motors used for hand movement control are servo motors, as depicted in Figure 2.

The servo motor in Figure 2 is a GWS Micro 2BBMG—micro servo with the following specifications [13]:

Torque: 5.40 kg-cm at 4.8 V, 6.41 kg-cm at 6.0 V Speed: 0.17 s/60° at 4.8 V, 0.14 s/60° at 6.0 V.

The GWS Micro 2BBMG—micro servo is an analog servo motor and does not have a built-in PID control algorithm.

Before configuring the system, we measured the time taken by the robot hand in Figure 1 to reach the target angle after applying a step input in order to verify the delay time. To verify the time it takes for the servo motor to move to the target angle, we used the AS5601 magnetic encoder along with an Arduino Mega. The AS5601 features a 12-bit resolution, offering a high-resolution output of 4096 PPR (Pulses Per Revolution). By utilizing the magnetic encoder, we measured the time and angle required for the servo motor to move from 0 degrees to the target angle of 60 degrees. Also, the transfer function of the control plant is derived using the measured step response.

### 2.2. System Design

This study builds upon the work of Masamichi Hayashi, Hiroshi Kogure, Kazuhide Ura, Masami Iwase, Teruyoshi Sadahiro, Shosiro Hatakeyama, and Eita Sawaguchi in their paper titled “Development of Zero-Phase-Tracking Man-Machine Interface with Electro-Mechanical Delay of Electromyogram.” The authors explored the application of Electro-Mechanical Delay (EMD) in EMG signals to synchronize human movements with machine responses. They implemented a Zero-Phase Error Tracking Control (ZPETC) system combined with a Nonlinear Delayed Auto-Regressive eXogenous (NDARX) model to predict wrist movements from EMG signals, reducing phase lag and improving system responsiveness.

Building on this prior work, our study combines PID control with ZPETC to further enhance the stability and response time of the myoelectric prosthesis control system. By integrating the EMD-based compensation approach from earlier research, we aim to minimize delay and develop a more responsive control system that enhances real-time performance. Additionally, we aim to validate the system’s effectiveness through real-world implementation, addressing the challenges identified in previous studies.

**Figure 3:**
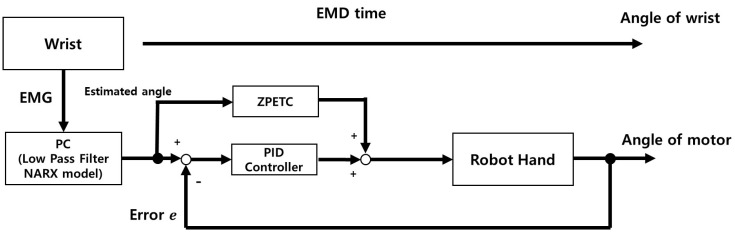
Block diagram of the control system used for the robotic hand. The system processes EMG signals obtained from the wrist through a PC-based NARX model with a low-pass filter to estimate the wrist angle. The estimated angle serves as input for two control strategies: the feedforward control (ZPETC) and the feedback control (PID controller). ZPETC compensates for phase delay by leveraging the EMD time, while the PID controller minimizes the error between the motor and wrist angles. The combined outputs of these controllers enable precise and responsive control of the robotic hand.

Figure 3 provides a block diagram of the control system used for the robotic hand. The system integrates Electro-Mechanical Delay (EMD) compensation into its processes to synchronize the user’s intent with the robotic hand’s response. First, EMG signals generated by wrist movements are captured and passed through a low-pass filter to remove noise and extract relevant features. These filtered signals are then processed by a PC-based NARX model, which estimates the wrist angle.

The estimated angle serves as the input for two control strategies: the feedforward control (ZPETC) and the feedback control (PID controller). ZPETC generates a target trajectory by leveraging the EMD time and ensures that the system tracks this trajectory without phase delay. Meanwhile, the PID controller minimizes the error between the motor’s output angle and the desired wrist angle by correcting for any deviations. The combined outputs of these controllers are sent to the robotic hand, ensuring precise and responsive control.

Finally, the system’s effectiveness is validated by comparing the motor angle with the actual wrist angle, assessing how accurately the robotic hand replicates the intended movement. The combination of ZPETC and the PID controller ensures fast, accurate, and stable operation by addressing both phase delay and real-time error correction.

### 2.3. EMG Signal Measurement

**Figure 4:**
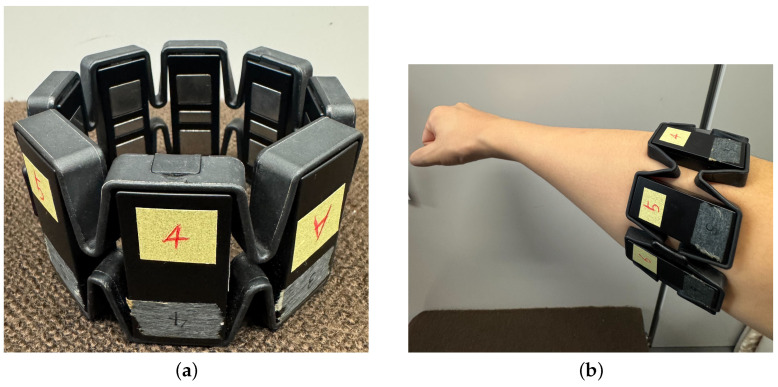
(**a**) Myo armband from Thalmic Labs. (**b**) Wearing position of Myo armband.

Measurement of the EMG signal uses the Myo armband in Figure 4a. The Myo armband is a wearable device equipped with eight EMG electrodes, a nine-axis inertial measurement device, and a transmission module. The sampling frequency is 200 Hz, and the data are transmitted to the outside using Bluetooth low energy (BLE) technology [14]. In this study, the four channels of the Myo armband are placed on the extensor carpi radialis longus, as shown in Figure 4b, and worn to acquire and use the EMG signal during the Palmar flexion and Dorsi flexion operation of the wrist.

This study was conducted using data from a single healthy 27-year-old male participant. The ultimate goal of this study is to develop a control system that is applicable to myoelectric prostheses. Since myoelectric prostheses are customized for individuals, the control system is designed to be calibrated by the user after development. Therefore, this study was conducted based on data from a single participant. To ensure reliability, data collection was performed under repeated conditions for the same participant. Also, this study utilized anonymized EMG signals that cannot identify specific individuals and collected data using non-invasive methods. The purpose of the data usage was limited to technical research, such as the development of an algorithm for estimating wrist angles. It has been confirmed that this study is outside the scope of ethical approval requirements under the Tokyo Denki University guidelines. Furthermore, this study adhered to ethical guidelines and ensured the protection of participants’ rights and privacy during data usage and processing.

**Figure 5:**
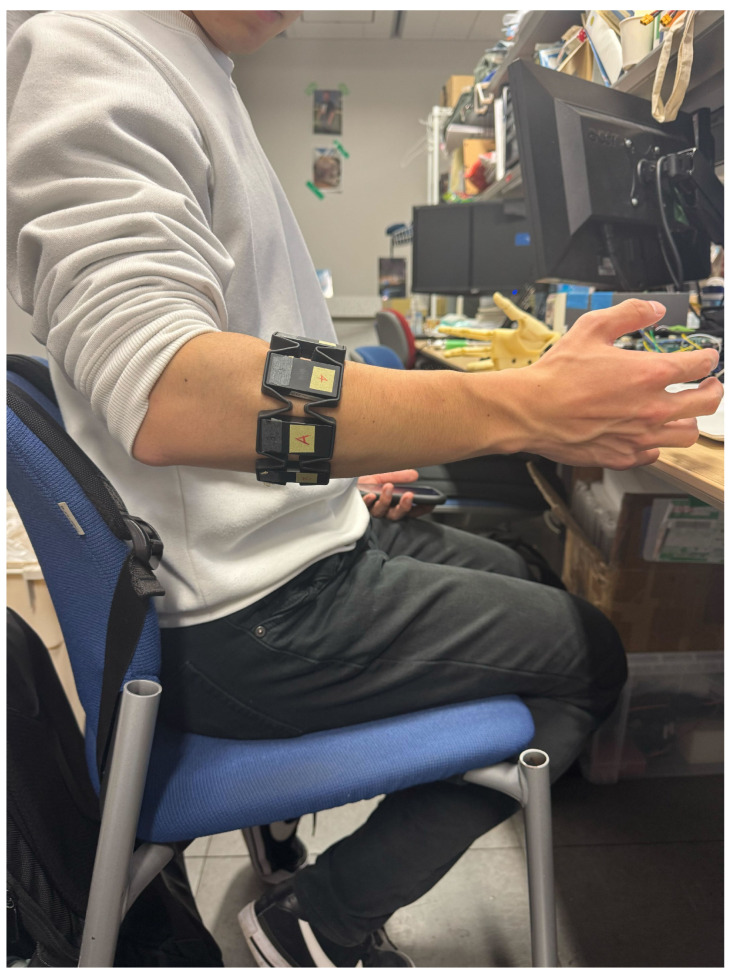
EMG measurement side view.

**Figure 6:**
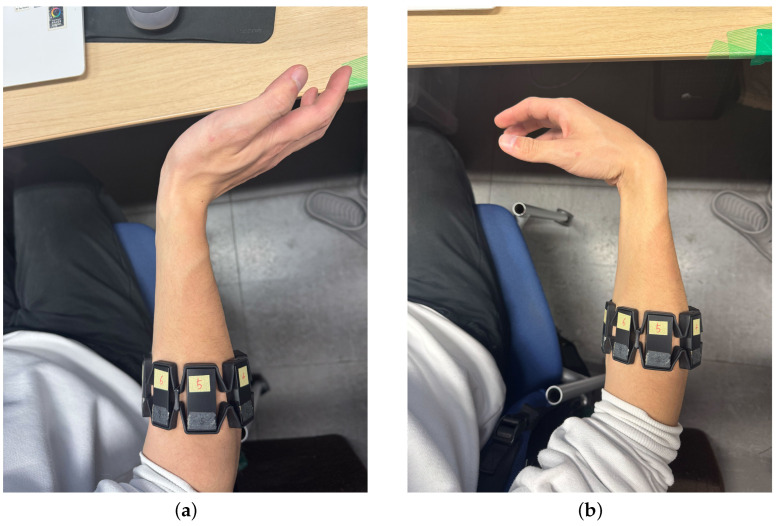
(**a**) Dorsi flexion. (**b**) Palmar flexion.

As shown in Figure 4b, the participant wore the Myo armband on the extensor carpi radialis longus of the right hand. During the experiment, the participant was seated comfortably in a chair with the arm positioned at approximately 90° relative to the torso, as illustrated in Figure 5. The participant alternated between Dorsi flexion (Figure 6a) and Palmar flexion (Figure 6b) movements, performing each movement for 5 s. For NARX model training data, wrist movements were performed at the participant’s maximum flexion angle (0° to −80° for Figure 6a and 0° to 80° for Figure 6b) with a controlled speed, completing 1–2 repetitions per movement within 5 s. Test data were collected by performing wrist movements with randomized angles, sequences, and speeds to evaluate the model’s generalization performance. The EMG signal contains high-frequency noise components. Therefore, high-frequency noise is removed using a low-pass filter (LPF). Specifically, muscle motor units associated with hand movements predominantly generate activation signals in the low-frequency band [15]. Additionally, a study utilizing Multivariate Variational Mode Decomposition (MVMD) [16] has demonstrated that the low-frequency components contain important motor information, and pattern recognition performance improves when extracting the low-frequency band. Based on this, the current study applied a low-pass filter with a cutoff frequency set at 5 Hz. The transfer function of the low-pass filter is represented by Equation (1). ω represents the angular frequency of the filter.

(1) $$\begin{array}{cc} G_{low-passfilter} & =\frac{\omega^{2}}{s^{2}+\sqrt{2}\omega s+\omega^{2}}\\ \omega & =2\pi f[\mathrm{rad}/s]\\ f & =5\;\mathrm{Hz} \end{array}$$

### 2.4. Wrist Angle Measurement

**Figure 7:**
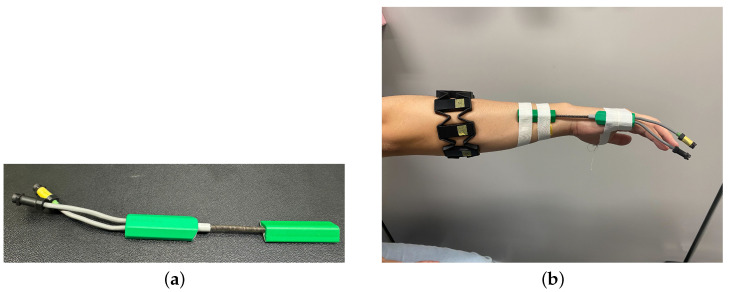
(**a**) SG65 Goniometer from Biometrics Ltd., a company based in Newport, United Kingdom. (**b**) Set up position of SG65.

The SG65 goniometer from Biometrics Ltd., shown in Figure 7a, was used to measure wrist angles. The SG65 is a wired, twin-axis electronic goniometer with a full measurement range of $\pm 180^{{}^{\circ}}$. This device offers an accuracy of $\pm 2^{{}^{\circ}}$ and a repeatability of $1^{{}^{\circ}}$. Considering human movement, an angular error of 2 degrees is generally negligible [17].

**Figure 8:**
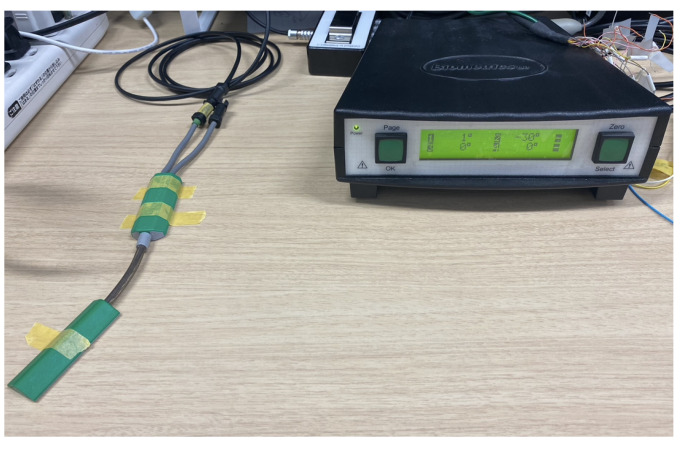
K800 Amplifier from Biometrics Ltd. (Newport, UK).

To measure wrist angles using the SG65, the joint of the SG65 was positioned on the wrist joint as shown in Figure 7b. The angle measured by the SG65 can be checked using the K800 Amplifier from the same company, as shown in Figure 8. The K800 amplifier has a sampling frequency of approximately 5 kHz [18,19]. The SG65 goniometer, when used with the K800 amplifier, specifies that 0V corresponds to $-180^{{}^{\circ}}$, 2V to $0^{{}^{\circ}}$, and 4V to $180^{{}^{\circ}}$. Using this information, the sensitivity of the sensor is calculated, as shown in Equations (2)–(6). The calculation result indicates that the sensitivity is approximately 0.0111V/degree.

The wrist joint angle measured by the SG65 is converted into voltage and transmitted to the PC. Therefore, it is necessary to confirm the correlation between voltage and joint angle. We measured the voltage and angle, increasing the angle from −90 degrees to 90 degrees in steps of 30 degrees. Additionally, when measuring the EMG signals and wrist angles simultaneously, the measurement was conducted by wearing both the Myo armband and the SG65 goniometer simultaneously, as shown in Figure 7a,b using the same method described in the EMG Signal Measurement section. Due to the difference in sampling frequencies between the two sensors, the SG65 data were configured to be collected only at the time points when EMG signals were recorded. This approach synchronized the sampling frequencies, making the analysis more convenient.

(2) $$\begin{array}{c} S=\frac{\Delta V}{\Delta \theta } \end{array}$$

where:

- *S*: Sensitivity (V/degree)
- ΔV: Change in output voltage from 0 to 4 (V)
- Δθ: Change in input angle from −180 to 180 (degrees)

Given:

(3) $$\begin{array}{c} \Delta V=4\;V \end{array}$$

(4) $$\begin{array}{c} \Delta \theta =360^{{}^{\circ}} \end{array}$$

Sensitivity is calculated as:

(5) $$\begin{array}{c} S=\frac{\Delta V}{\Delta \theta }=\frac{4\;V}{360^{{}^{\circ}}}=0.0111\;V/\mathrm{degree} \end{array}$$

Therefore, the sensitivity of the SG65 goniometer is approximately:

(6) $$\begin{array}{c} S=0.0111\;V/\mathrm{degree} \end{array}$$

### 2.5. NARX Model (Nonlinear AutoRegressive eXogenous Model)

To control a servo motor using EMG signals, it is essential to understand the correlation between wrist angle and EMG signals. Furthermore, since EMG signals exhibit nonlinearity, a model with coefficients that vary depending on the output is required. To model this correlation, we propose the Nonlinear AutoRegressive eXogenous (NARX) model. The NARX model is well-suited for capturing the nonlinear characteristics of EMG signals. Additionally, NARX models are ideal for time-series prediction, as the output depends on past output values (autoregressive property) and exogenous inputs. Therefore, the NARX model is appropriate for deriving estimated angles from EMG in real-time to be used as the reference trajectory for ZPETC.

To understand the NARX model, it is necessary to first comprehend the ARX (AutoRegressive with eXogenous) model, its foundational form. The ARX model represents the output of a dynamic system as a linear combination of past output values and external input values. The basic equation of the ARX model is defined as follows:

(7) $$\begin{array}{cc} y\left(k\right)+a_{1}y\left(k-1\right)+a_{2}y\left(k-2\right)+\cdots +a_{n_{a}}y\left(k-n_{a}\right) & =\\ b_{1}u\left(k-1\right)+b_{2}u\left(k-2\right)+\cdots +b_{n_{b}}u\left(k-n_{b}\right) \end{array}$$

y(k): Output at time *k*, e.g., wrist angle (V).
u(k): Input at time *k*, e.g., EMG signal amplitude (V).
$a_{i}$: Coefficients of the autoregressive (output) terms.
$b_{i}$: Coefficients of the exogenous (input) terms.
$n_{a}$: Number of past output data points (output lag order).
$n_{b}$: Number of past input data points (input lag order).

In Equation (7), $n_{a}$ and $n_{b}$ are set to 2. The order selection for the ARX and NARX models will be described in detail in the subsequent Determination of the NARX Model Orders $n_{a}$, $n_{b}$, and Interval *m* section. Additionally, since the input for the EMG signals in this experiment consists of eight channels, u(k) is replaced with $u_{i}$ (i=1,⋯,8) and $b_{i}$ with $b_{ij}$ (j=1,2). The modified equation can be expressed as follows:

(8) $$\begin{array}{cc} y(k)= & -a_{1}y\left(k-1\right)-a_{2}y\left(k-2\right)\\ \; & +b_{11}u_{1}\left(k-1\right)+b_{12}u_{1}\left(k-2\right)\\ \; & +\cdots +b_{81}u_{8}\left(k-1\right)+b_{82}u_{8}\left(k-2\right) \end{array}$$

Equation (8) represents the fundamental ARX model equation used in this study. The NARX model extends this equation to accommodate nonlinear signals.

The NARX model divides the dataset of wrist angles (output) and electromyography (EMG) signals (input) into *m* segments based on the output values and applies a separate ARX model to each segment to capture nonlinearity. Here, *m* represents the number of divisions within the output range, enabling a detailed analysis of the data characteristics within each segment.

In this study, *m* is set to 4, and the rationale for this choice will be discussed in detail in the Determination of the NARX Model Orders $n_{a}$, $n_{b}$, and Interval *m* section.

(9) $$\begin{array}{cc} \mathrm{For}\;\mathrm{segment}\;m\left(m=1,\cdots ,4\right):y_{m}\left(k\right)= & -a_{11,m}y_{m}\left(k-1\right)-a_{12,m}y_{m}\left(k-2\right)\\ \; & +b_{11,m}u_{1,m}\left(k-1\right)+b_{12,m}u_{1,m}\left(k-2\right)\\ \; & +\cdots +b_{81,m}u_{8,m}\left(k-1\right)+b_{82,m}u_{8,m}\left(k-2\right) \end{array}$$

The coefficients for each segment *m* in (9) can be reorganized as coefficient functions $\alpha_{1}\left(y\right),\alpha_{2}\left(y\right),\beta_{11}\left(y\right),\cdots ,\beta_{82}\left(y\right)$, resulting in the following MISO-NARX model equation:

(10) $$\begin{array}{cc} y(k)= & -\alpha_{1}\left(y\right)y\left(k-1\right)-\cdots -\alpha_{n_{a}}\left(y\right)y\left(k-n_{a}\right)\\ \; & +\beta_{11}\left(y\right)u\left(k-1\right)+\cdots +\beta_{1n_{b}}\left(y\right)u\left(k-n_{b}\right)\\ \; & +\beta_{21}\left(y\right)u\left(k-1\right)+\cdots +\beta_{2n_{b}}\left(y\right)u\left(k-n_{b}\right)\\ \; & \;\;\;\;\;\;\;\;\;\;\;\;\;\;\;\;\;\;\;\;\;\;\;\;\;\vdots \\ \; & +\beta_{81}\left(y\right)u\left(k-1\right)+\cdots +\beta_{8n_{b}}\left(y\right)u\left(k-n_{b}\right) \end{array}$$

Using the constructed MISO-NARX model (10), wrist joint angles can be estimated from electromyography (EMG) signals. For the estimation, coefficients such as $a_{1},a_{2},\cdots ,b_{82}$ in (8) need to be determined. These coefficients are represented as a coefficient vector $\theta_{m}$. This can be expressed as Equation (11). The corresponding calculation is described using the least squares method in Equation (12).

The inputs and outputs used in Equation (12) are represented by Equations (13) and (14), respectively. Here, $X_{m}$ represents the input data in segment *m*, and $Y_{m}$ represents the output data in segment *m*.

(11) $$\begin{array}{c} \theta_{m}={\left[\begin{array}{ccccc} a_{1,m} & a_{2,m} & b_{11,m} & \cdots & b_{82,m} \end{array}\right]}^{T} \end{array}$$

(12) $$\begin{array}{c} \theta_{m}={\left(X_{m}^{T}X_{m}\right)}^{-1}X_{m}^{T}Y_{m} \end{array}$$

(13) $$\begin{array}{c} X_{m}=\left[\begin{array}{cc} -y_{m}\left[k_{1}-1\right] & -y_{m}\left[k_{1}-2\right]\\ \vdots & \vdots \\ -y_{m}\left[k_{N}-1\right] & -y_{m}\left[k_{N}-2\right] \end{array}\right.\\ \left.\begin{array}{ccc} u_{11}\left[k_{1}-1\right] & \cdots & u_{82}\left[k_{1}-2\right]\\ \vdots & & \vdots \\ u_{11}\left[k_{N}-1\right] & \cdots & u_{82}\left[k_{N}-2\right] \end{array}\right] \end{array}$$

(14) $$\begin{array}{c} Y_{m}={\left[\begin{array}{cccc} y_{m}\left[k_{1}\right] & y_{m}\left[k_{2}\right] & \cdots & y_{m}\left[k_{N}\right] \end{array}\right]}^{T} \end{array}$$

Using the same method, the coefficient functions $\alpha_{1}\left(y\right),\alpha_{2}\left(y\right),\beta_{11}\left(y\right),\cdots ,\beta_{82}\left(y\right)$ can be determined, and the MISO-NARX model (10) can be constructed.

### 2.6. Ridge Regression

When constructing the MISO-NARX model, increasing the complexity of the model raises the likelihood of overfitting to the training data. In this study, we use EMG signals from eight channels, each of which is related to wrist movements. Since the signals from each channel are highly correlated with each other, the risk of overfitting increases even more. Highly correlated data can significantly increase the variance of the model coefficients, causing the model to become overly sensitive to specific data points.

To address this issue, we propose applying ridge regression. Ridge regression works by incorporating L2 regularization, which limits the magnitude of the model’s coefficients, thereby adjusting the influence of each variable. This prevents the model from assigning excessively large values to the coefficients, which helps improve the generalization performance of the model and prevents overfitting [12]. In this study, we set the L2 parameter λ to 0.01. Let the coefficient vector of ridge regression be denoted as ${\hat{\theta }}_{k}$, and the calculation follows the equation below.

(15) $$\begin{array}{c} {\hat{\theta }}_{k}={\left(X_{k}^{T}X_{k}+\lambda I\right)}^{-1}X_{k}^{T}Y_{k} \end{array}$$

### 2.7. Zero Phase Error Tracking Control (ZPETC)

Zero Phase Error Tracking Control (ZPETC) is a control algorithm used in control systems to enhance tracking performance in discrete-time systems. ZPETC minimizes discrepancies between input and output by eliminating the system’s phase error. To achieve phase error elimination, the inverse transfer function of the system is calculated and applied to the control input, allowing the output to accurately follow the reference signal.

In our work, we use the wrist angle estimated by the NARX model as the reference signal, and ZPETC is employed to control the input for the servo motor. By compensating for the phase error in the servo motor, this approach enables the motor to reach the target angle more quickly and accurately.

Assume that the control plant is of the form as shown in (16), and the plant is either stable or stabilized. Here, $z^{-1}$ is the delay operator, the numerator polynomial is denoted as $B[z^{-1}]$, and the denominator polynomial is denoted as $A[z^{-1}]$. When $B[z^{-1}]$ has unstable zeros, factorizing it yields the expression shown in (19). Here, $B^{-}$ is a monic polynomial of degree *s* that includes both unstable and marginally stable zeros, and $B^{+}$ is a polynomial of degree m−s that includes only stable zeros. A monic polynomial is a polynomial where the highest degree term has a coefficient of 1. In this case, the equation for ZPETC is expressed as shown in (20). Here, $B^{-}\left[1\right]$ represents the steady-state gain of $B^{-}\left[z^{-1}\right]$. The inverse polynomial used to compensate for the unstable zeros is shown in (21).

*z*^−1^: Delay operator representing a one-step time delay, e.g., *z*^−1^*y*[*k*] = *y*[*k* − 1].
*B*[*z*^−1^]: Numerator polynomial of the transfer function, defined as *B*[*z*^−1^] = *b*_0_ + *b*_1_*z*^−1^ + ⋯ + *b_m_z*^−*m*^, representing the system’s response to input.
*A*[*z*^−1^]: Denominator polynomial of the transfer function, defined as *A*[*z*^−1^] = 1 + *a*_1_*z*^−1^ + ⋯ + *a*_*n*_*z*^−*n*^, governing the system stability.
*B*^−^[*z*^−1^]: Unstable or marginally stable zeros of *B*[*z*^−1^], requiring compensation in ZPETC.
*B*^+^[*z*^−1^]: Stable zeros of *B*[*z*^−1^], not requiring compensation in ZPETC.
*c*_1_: Ratio *b*_0_/*b*_1_, representing the steady-state gain, used for compensating *B*^−^[*z*^−1^].

(16) $$\begin{array}{c} G\left[z^{-1}\right]=\frac{z^{-d}B\left[z^{-1}\right]}{A[z^{-1}]} \end{array}$$

(17) $$\begin{array}{c} B\left[z^{-1}\right]=b_{0}+b_{1}z^{-1}+\cdots +b_{m}z^{-m},\;b_{0}\neq 0 \end{array}$$

(18) $$\begin{array}{c} A\left[z^{-1}\right]=1+a_{1}z^{-1}+\cdots +a_{n}z^{-n} \end{array}$$

(19) $$\begin{array}{c} B\left[z^{-1}\right]=B^{-}\left[z^{-1}\right]B^{+}\left[z^{-1}\right] \end{array}$$

(20) $$\begin{array}{c} G_{ZPETC}\left[z^{-1}\right]=\frac{A\left[z^{-1}\right]B^{-*}\left[z^{-1}\right]}{B^{+}\left[z^{-1}\right]{\left(B^{-}\left[1\right]\right)}^{2}} \end{array}$$

(21) $$\begin{array}{c} B^{-*}\left[z^{-1}\right]=z^{-s}B^{-}\left[z\right] \end{array}$$

(22) $$\begin{array}{cc} G[z] & =\frac{b_{0}z+b_{1}}{z^{2}+a_{1}z+a_{2}}\\ \Leftrightarrow G[z^{-1}] & =\frac{\left(z^{-1}+c_{1}\right)b_{1}z^{-1}}{a_{2}z^{-2}+a_{1}z^{-1}+1} \end{array}$$

Equation (22) can be expressed in the form of (16) as follows.

(23) $$\begin{array}{c} A\left[z^{-1}\right]=a_{2}z^{-2}+a_{1}z^{-1}+1 \end{array}$$

(24) $$\begin{array}{c} B^{-}\left[z^{-1}\right]=\left(z^{-1}+c_{1}\right) \end{array}$$

(25) $$\begin{array}{c} B^{+}\left[z^{-1}\right]=b_{1} \end{array}$$

(26) $$\begin{array}{c} B^{-*}\left[z^{-1}\right]=z^{-1}B^{-}\left[z\right]=z^{-1}\left(c_{1}+z\right) \end{array}$$

By substituting Equations (23)–(26) into (20), the zero phase error tracking controller (ZPETC) can be obtained as follows.

(27) $$\begin{array}{c} G_{ZPETC}\left[z^{-1}\right]=\frac{A\left[z^{-1}\right]z^{-1}\left(c_{1}+z\right)}{b_{1}{\left(c_{1}+1\right)}^{2}} \end{array}$$

## 3. Results

### 3.1. System Delay Verification

The results of applying a 60-degree step input to the motor in Figure 2, used in the robotic hand system, are shown in Table 1. As shown in Table 1, when a target value of 60 degrees is applied at 0 s, the system reaches approximately 60 degrees and stabilizes at around 0.423 s on average. Based on this, the delay time for the human–machine interface in the robotic hand system is considered to be approximately 0.423 s.

**Figure 9:**
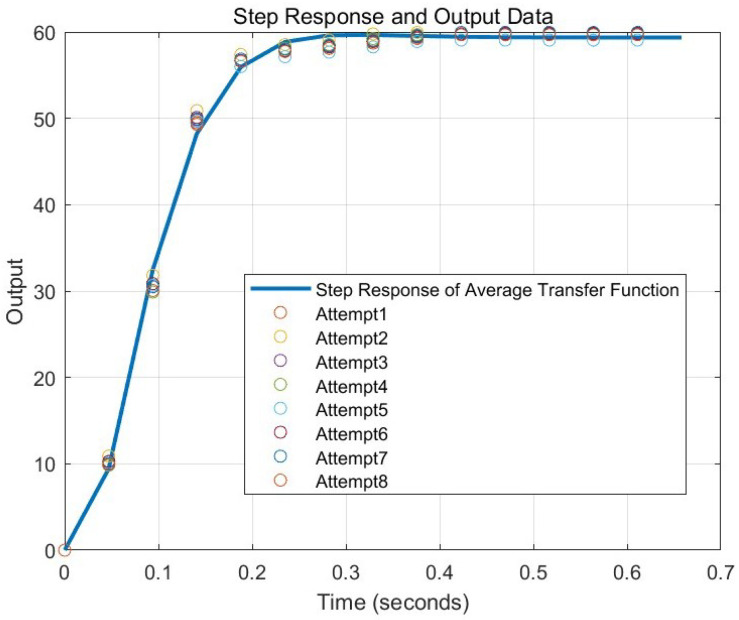
Step response of discretized transfer function and output data.

Additionally, second-order transfer functions were derived from each dataset, and the average transfer function was calculated. The resulting average transfer function is presented in (28). The discretized transfer function, which was obtained for use in the ZPETC, can be expressed as shown in (29). The discretization was performed using the Zero-Order Hold (ZOH) method. The step response of (29) and the data points from Table 1 are plotted in Figure 9. Equation (29) follows the form of (22), and the coefficients of the transfer function are summarized in Table 2.

(28) $$G\left(s\right)=\frac{-5.673s+423.1}{s^{2}+35.55s+427.6}$$

(29) $$G\left(z\right)=\frac{0.1573z+0.2637}{z^{2}-0.7626z+0.1881}$$

### 3.2. EMG Measurement

**Figure 10:**
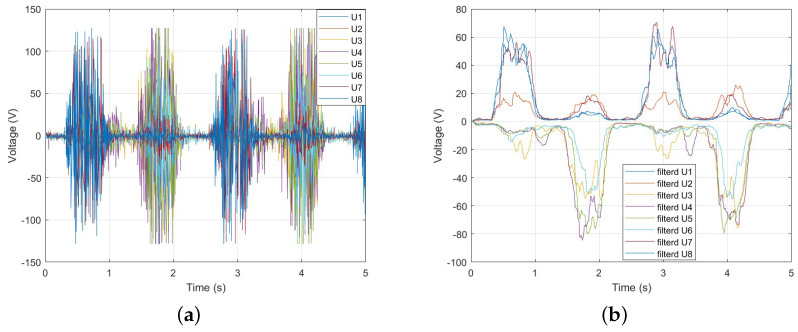
(**a**) Measured EMG signal by Myo armband. (**b**) low-pass filtered EMG signal.

Figure 10 shows the forearm EMG signals during Palmar flexion and Dorsiflexion of the wrist. In Figure 10a, U1 to U8 represent the eight-channel EMG signals measured using the Myo armband. To remove high-frequency noise and utilize the low-frequency components, the EMG signals processed with a low-pass filter are shown in Figure 10b. As seen in Figure 10b, specific EMG signals are activated according to the wrist movement.

### 3.3. Bluetooth Latency of Myo Armband

The Myo armband in Figure 4a transmits EMG signals through Bluetooth communication. To use the wireless Myo armband and the wired goniometer simultaneously, it is necessary to examine the communication delay associated with the wireless communication. The timestamp provided within the Myo armband is measured in Unix time. Therefore, by comparing the timestamp at which the EMG signal is obtained with the Unix time when the EMG is received by the PC program, the communication delay can be identified. The measurement results are shown in Table 3. The average delay time was found to be 0.001078 s, with a minimum delay time of 0.000272 s and a maximum delay time of 0.034995 s This result will be mentioned again in the verification of EMD discussed later.

### 3.4. Wrist Angle Measurement Results

**Figure 11:**
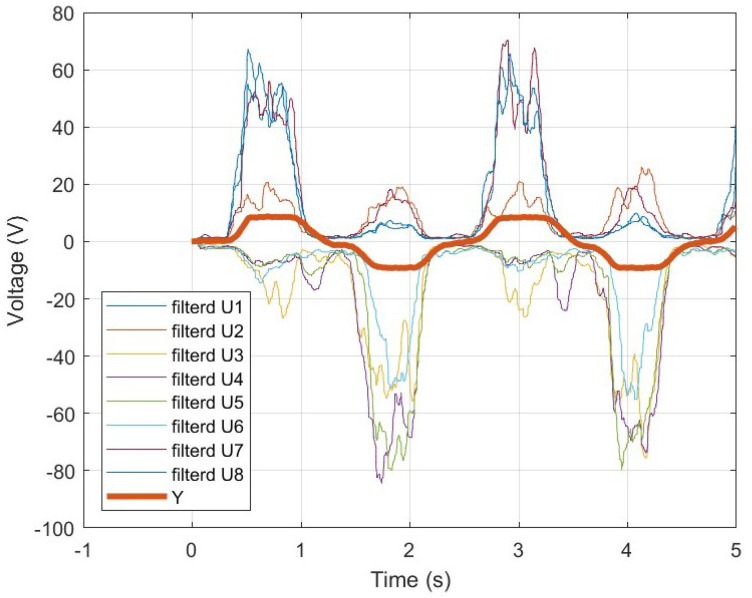
Wrist angle by goniometer.

As illustrated in Figure 7b, wrist angles can be measured simultaneously with EMG signals, and their combined representation is shown in Figure 11. In this figure, Y denotes the wrist angle measured in units of [V]. Since the wrist angle values are considerably smaller compared to the EMG signals, they have been amplified tenfold for better visualization. Based on the relationship between angle and voltage, which will be discussed later, the voltage corresponding to an angle of 0 degrees is 2.01 V. To align the baseline to zero, 2.01 V was subtracted from all angle values. This adjustment was applied solely to improve the clarity of the graph, enabling an easy comparison between EMG signals and wrist angles, and it does not affect the calculations.

The measured angle is represented as a voltage value, making it necessary to examine the correlation between angle and voltage. Table 4 presents the voltage corresponding to each angle.

Additionally, the results in Table 4 can be expressed as in (23), where *y* represents the voltage and *x* represents the angle.

(30) $$\begin{array}{c} y=0.0112x+2.01 \end{array}$$

#### Verification of EMD

**Figure 12:**
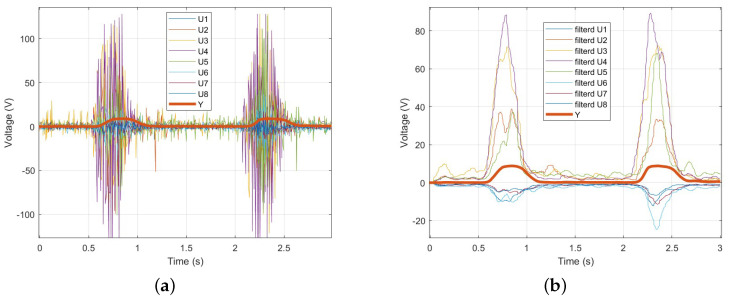
(**a**) Raw EMG signals and wrist angles. (**b**) Low-pass filtered EMG signals and wrist angles.

**Figure 13:**
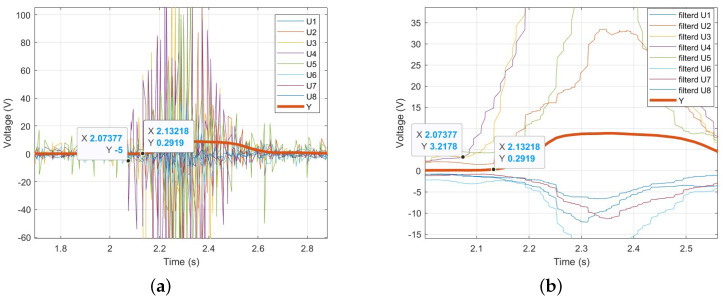
(**a**) Enlarged view of raw EMG signals and wrist angles. (**b**) Enlarged view of low-pass filtered EMG signals and wrist angles.

To verify EMD, EMG signals and wrist angles were simultaneously measured, and the time at which changes occurred was recorded and compared. Figure 12a shows the raw, unfiltered EMG signals and wrist angles, while Figure 12b presents the EMG signals and wrist angles after applying a 5 Hz low-pass filter. In Figure 12, it can be observed that the onset of wrist angle movement occurs later than the EMG signal. This delay represents the EMD. Additionally, Figure 13 is an enlarged view of Figure 12. In Figure 13a, 2.07377 s marks the time when the EMG signal starts to appear, and 2.13218 s marks the time when the wrist angle begins to change. The difference between these two times is 0.05841 s. In Figure 13b, the low-pass filtered EMG signal also allows for measuring the EMD within the same interval. This result represents the time without the Bluetooth communication delay of the Myo armband, as confirmed in Section 2.3. Therefore, in real-time control, the time obtained by subtracting the communication delay from the EMD time will be available. As a result, approximately 0.058138 s at maximum, 0.023415 s at minimum, and an average of approximately 0.057332 s of EMD time can be used to improve the system’s responsiveness.

### 3.5. Determination of the NARX Model Orders $n_{a}$, $n_{b}$, and Interval m

Various simulations were conducted to determine the orders $n_{a}$, $n_{b}$ of the NARX model and the interval *m* for applying the ARX model. In the initial phase of the simulation process, careful attention was given to establishing the orders of the ARX and NARX models. The model order is a crucial parameter, as it defines the extent to which the model depends on past data to make accurate predictions. This dependency is essential in determining the complexity and predictive performance of the model.

To systematically determine the optimal order, the Akaike Information Criterion (AIC) values for the ARX model were calculated. The AIC provides a mathematical framework for balancing model fit and complexity by penalizing excessive parameters, ensuring the model remains both accurate and parsimonious. Two separate training data sets were employed to train the ARX and NARX models. The training process was designed to ensure robustness across different scenarios by utilizing diverse data inputs.

**Figure 14:**
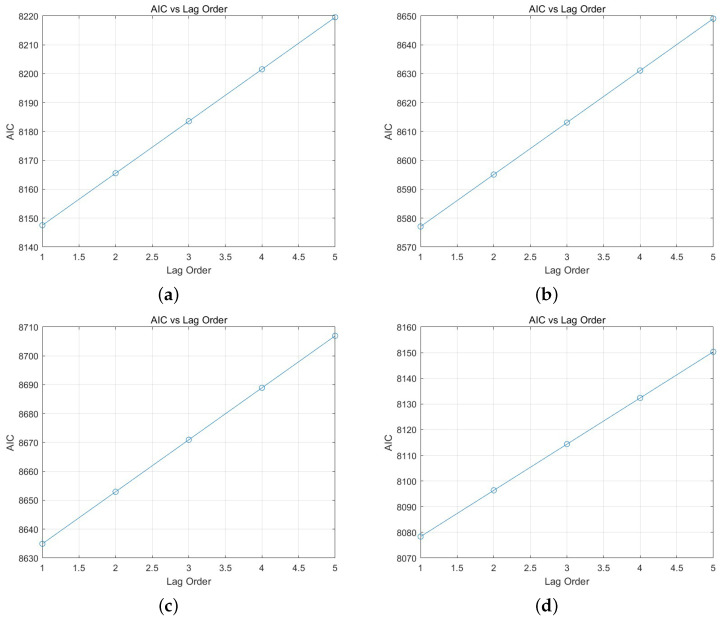
(**a**) The AIC results for Dataset 1. (**b**) The AIC results for Dataset 2. (**c**) The AIC results for Dataset 3. (**d**) The AIC results for Dataset 4.

In addition, a distinct test data set, which was not included in the training process, was used to rigorously evaluate the generalization performance of the models and confirm their ability to predict unseen data accurately. The results of the AIC calculations, which were applied to the test data set, are visually summarized and presented in Figure 14. This comparison highlights the performance differences across various model orders, providing insight into the selection of the most appropriate configuration. The formula used for AIC calculation is provided below for reference, emphasizing its role in guiding the decision-making process and optimizing model selection.

Through this comprehensive approach, the simulation results provide a strong foundation for determining the most effective model configuration for ARX and NARX applications.

(31) AIC=2k−2ln(L)

where:

- *k*: Number of parameters in the model
- *L*: Maximum likelihood of the model

**Figure 15:**
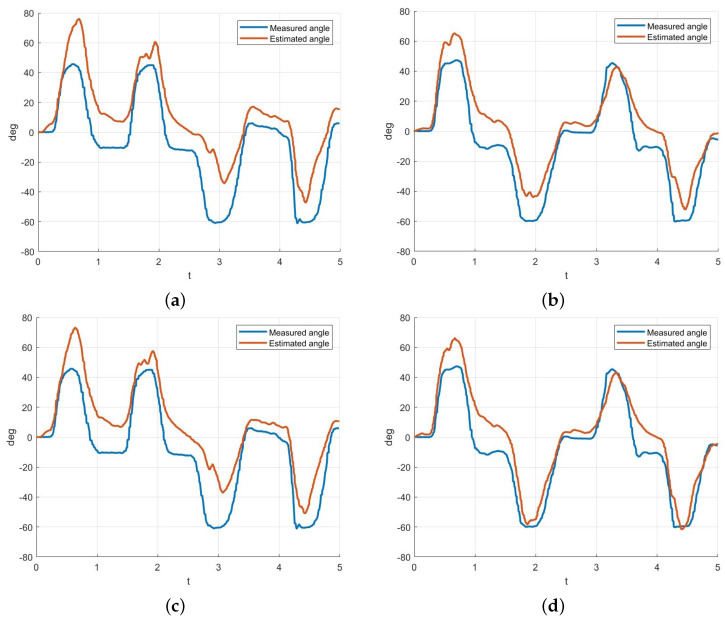
(**a**) The ARX estimation results for Dataset 1 with an order of 1 are presented below. (**b**) The ARX estimation results for Dataset 2 with an order of 1 are presented below. (**c**) The ARX estimation results for Dataset 1 with an order of 2 are presented below. (**d**) The ARX estimation results for Dataset 2 with an order of 2 are presented below.

From the results in Figure 14, it can be observed that the AIC value is the lowest when the order is 1 for all datasets. However, an order of 1 in the ARX and NARX models implies that the next value is predicted solely based on the current data. Therefore, the results of the ARX model with $n_{a},n_{b}=1$ and $n_{a},n_{b}=2$ will be examined. The estimation results are presented in Figure 15 and Table 5. Figure 15a,b represents the NARX model with an order of 1, while (c, d) shows the model with an order of 2. By comparing the graphs, it can be observed that negative angles are generated more accurately when the order is set to 2. According to the results in Table 5, it was observed that the estimation RMSE of the ARX model with an order of 2 was approximately 3 degrees smaller for the first dataset and about 1.7 degrees smaller for the second dataset compared to an order of 1. Furthermore, it was confirmed that the output for negative angles was smaller with an order of 1 compared to an order of 2. These results are considered to be highly critical for controlling the robotic hand. Therefore, in this study, the order of the ARX and NARX models was set to 2.

The following describes the process of setting the interval *m* for the NARX model.

After setting the order of the NARX model to 2, the interval *m* was incrementally increased to 2, 3, 4, and 5, and the results were compared. m=1 was excluded because it corresponds to the ARX model. Additionally, m≥6 was excluded from the comparison since the RMSE continued to increase beyond this point.

From Table 6, it is observed that the RMSE is the smallest when m=4, while it increases again for m=5. Therefore, in this study, *m* was set to 4.

### 3.6. Estimation of Wrist Angle by NARX

**Figure 16:**
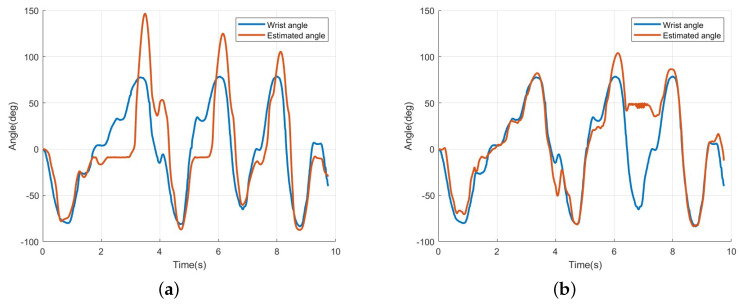
(**a**) The estimation result of wrist angle using EMG, 4ch SISO-NARX model. (**b**) The estimation result of wrist angle using EMG, 4ch MISO-NARX model.

**Figure 17:**
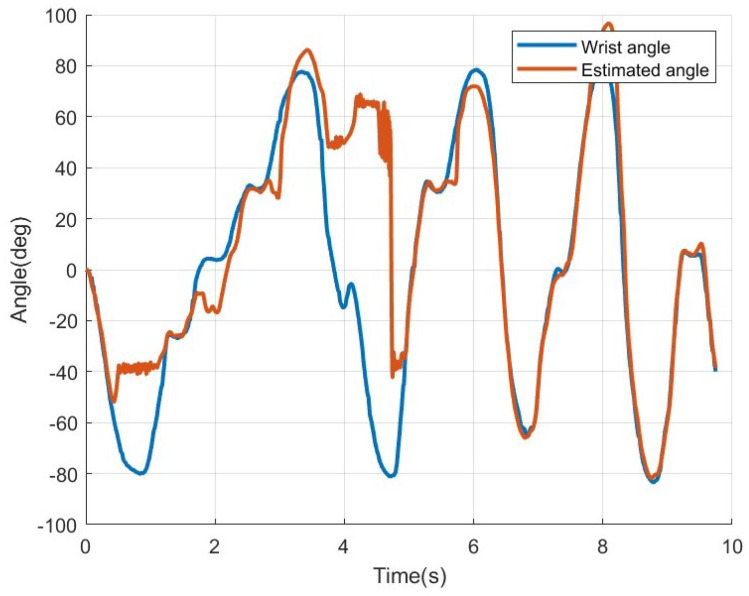
The estimation result of wrist angle using EMG, 8ch MISO-NARX model.

To verify the effectiveness of the proposed MISO-NARX model using eight-channel EMG signals, the four-channel SISO-NARX model and the four-channel MISO-NARX model from previous studies were first implemented, and their results were evaluated. Figure 16a presents the results of the four-channel SISO-NARX model, while Figure 16b illustrates the results of the four-channel MISO-NARX model. For the case shown in Figure 16a, the RMSE of angle estimation was approximately 32.70 deg. In contrast, Figure 16b shows that the RMSE of angle estimation was about 29.15 deg. Additionally, excessive errors were observed around the 6–7 s mark. From Figure 16, it can be confirmed that the RMSE of the MISO-NARX model decreased by approximately 3.55 deg, demonstrating the validity of the MISO model. Furthermore, the results of the MISO-NARX model extended to an eight-channel EMG signal configuration are shown in Figure 17. The RMSE in Figure 17 was about 33.72 deg, indicating lower estimation accuracy compared to Figure 16. However, for the 0–5 s interval, the estimation error was approximately 46.55 deg, and for the 5–10 s interval, the estimation error was around 7.09 deg, revealing that significant errors occurred in specific sections. Additionally, the occurrence of excessive errors in specific sections of the MISO-NARX model highlights the necessity of introducing ridge regression.

### 3.7. Ridge Regression

**Figure 18:**
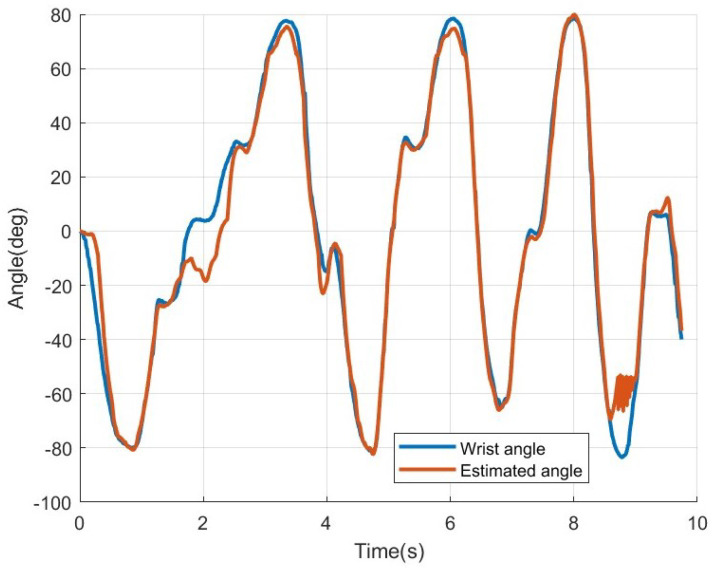
Wrist angle estimated using a 8ch MISO-NARX model with ridge regression.

**Figure 19:**
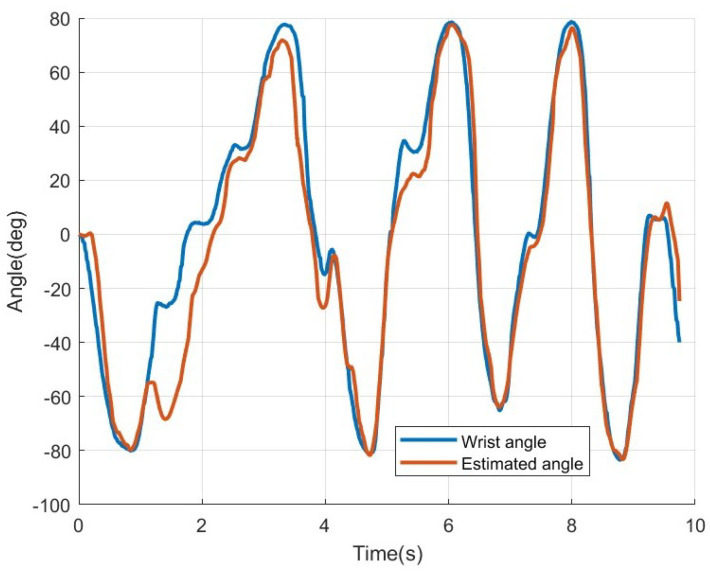
Wrist angle estimated using a 4ch MISO-NARX model with ridge regression.

Figure 18 shows the results of applying ridge regression to the eight-channel MISO-NARX model from Figure 17. When ridge regression is applied, the RMSE improves to 7.80 deg, showing an improvement of approximately 25.92 deg compared to before the application. Additionally, the excessive errors observed in specific sections were resolved. Furthermore, Figure 19 illustrates the results of applying ridge regression to the four-channel MISO-NARX model shown in Figure 16b. In this case, the RMSE was approximately 13.85 deg; an improvement of about 15.3 deg compared to before the application. Therefore, it was confirmed that applying ridge regression to the MISO-NARX model prevents overfitting and improves estimation performance.

### 3.8. ZPETC+PID Controller

#### 3.8.1. Stability of Control Systems

**Figure 20:**
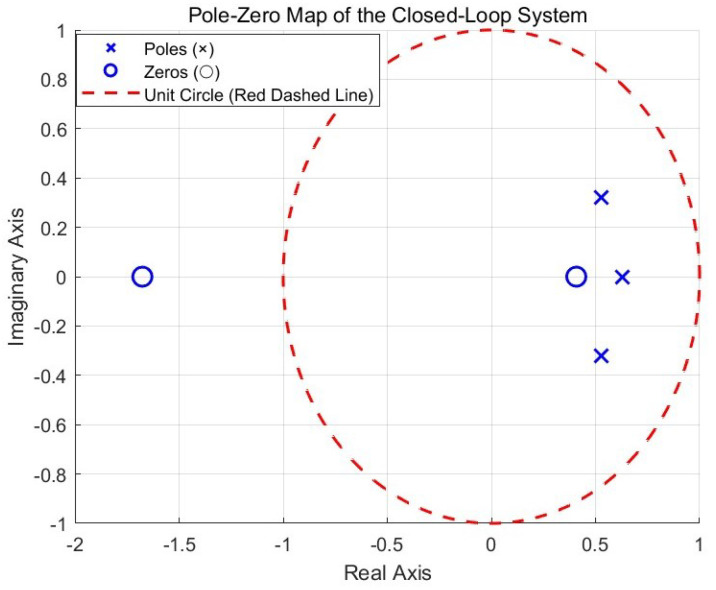
Pole-zero map of the closed-loop system.

In a control system combining ZPETC and PID control, ZPETC acts as a feedforward control, which does not affect the stability of the system. Therefore, the stability between the controlled plant and the PID control is examined. The transfer function of the plant is the same as (29), and the gains of the PID control are shown in Table 7. From Figure 20, it can be concluded that the closed-loop system is stable, as all poles are located inside the unit circle.

#### 3.8.2. Performance Simulation of the ZPETC+PID Controller

The GWS Micro 2BBMG servo motor, used in the existing robotic hand system, operates without any control system applied. This study validates the effectiveness of a two-degrees-of-freedom servo control system that combines the ZPETC+PID controller with this uncontrolled servo motor through simulation. The step duration was set to 0.047 s, and the target angle was set to 60 degrees. Additionally, since the ZPETC+PID controller application considers Electro-Mechanical Delay (EMD), it is assumed that the estimated angle is derived from EMG signals obtained approximately 2 steps prior, which is close to the maximum EMD value. Furthermore, as the control target differs from that in the study by Hayashi et al., a new benchmark is established. In Section 3.1, the average delay time of the servo motor is set to approximately 0.423 s. Consequently, this benchmark of 0.423 s will be used to evaluate the speed changes. The ideal outcome assumes a delay time of 0 s, meaning that the servo motor’s angle would immediately reach 60 degrees as soon as a 60-degree step input is applied. The accuracy of operation will be assessed based on the angular error when a 60-degree step input is provided.

**Figure 21:**
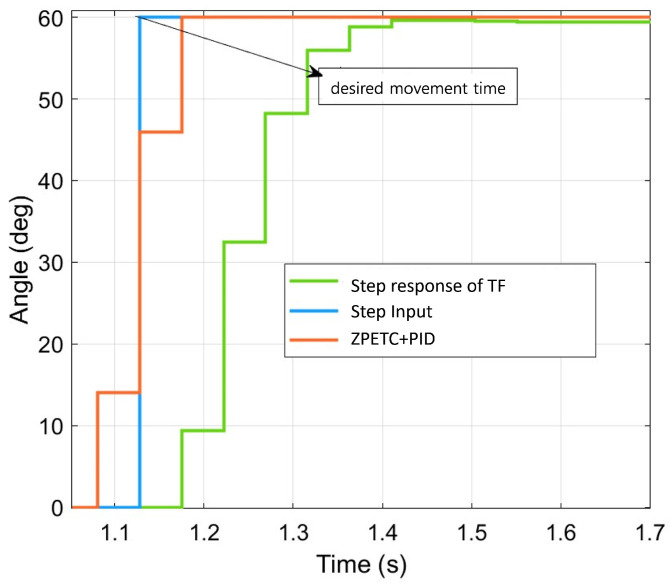
Simulation results when step input is applied to the system transfer function and the system transfer function with ZPETC+PID controller.

In Figure 21, the desired timing for the servo motor to reach the target angle of 60 degrees is approximately 1.128 s, which corresponds to the time the step input reaches 60 degrees. The step response of the transfer function (TF) represents the output when a step input is applied to the system, as shown in Figure 9. Here, the settling time is approximately 1.551 s, indicating a delay of about 0.423 s beyond the target time to reach the target angle of 60 degrees.

When the ZPETC+PID controller, which considers EMD, is applied, the time to reach the target angle is approximately 1.175 s, showing a delay of only about 0.047 s beyond the target time. The simulation results confirm that applying the ZPETC+PID controller reduces the time to reach the target angle by 0.376 s compared to the previous setup.

**Figure 22:**
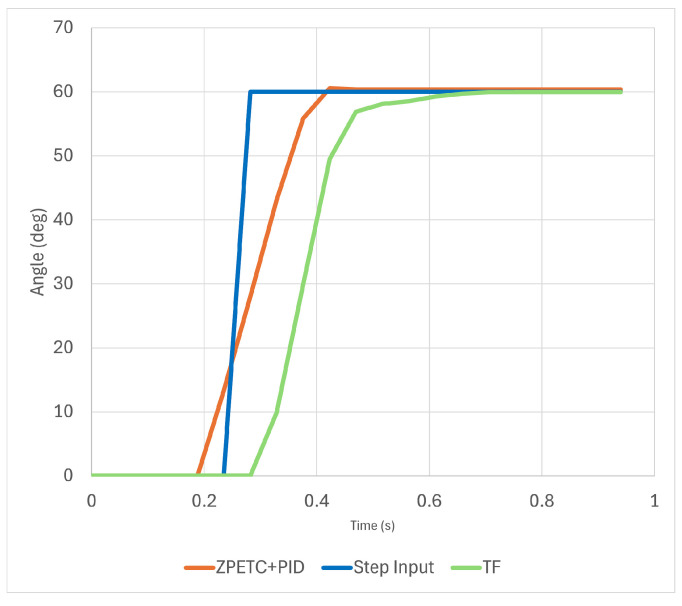
Output results when the ZPETC+PID controller is applied to the robotic hand system.

Figure 22 shows the results when the actual servo motor was operated under the same preconditions. In the actual system, when the ZPETC+PID controller was not applied, the time to reach the target was delayed by approximately 0.428 s. With the ZPETC+PID controller applied, the delay was reduced to approximately 0.188 s, reaching the target about 0.240 s faster than without the controller.

## 4. Discussion

### 4.1. Summary and Interpretation of Results

This study builds upon the research by Hayashi et al., aiming to resolve challenges encountered during practical implementation. For this purpose, the following hypotheses were formulated and verified.

The first hypothesis posits that switching from a four-channel wired EMG sensor to an eight-channel wireless EMG sensor may introduce communication delays, and EMD (Electro-Mechanical Delay) could still be effectively utilized in such an environment. Studies using the same EMG sensors do not mention communication delays when using wireless sensors [10,11]. While communication delay may not be critical in studies focusing on achieving accurate motions, it is considered an important verification in this study, as it is necessary to improve both the accuracy of motion and the system’s responsiveness. In Section 2.3, *Bluetooth latency of Myo armband*, the communication delay was measured, showing a minimum delay of 0.000272 s, a maximum delay of 0.034995 s, and an average delay of 0.001078 s. Additionally, in Verification of EMD Section, *Verification of EMD*, an EMD of approximately 0.05841 s was confirmed by analyzing the delay between EMG signal generation and wrist angle change. Relating these results to the observed communication delay, it was calculated that EMD could still be utilized effectively, with values ranging from a minimum of 0.023415 s to a maximum of 0.058138 s, and an average of 0.057332 s. These findings suggest that even with a wireless EMG sensor, EMD can be leveraged to enhance system responsiveness.

The second hypothesis states that the eight-channel MISO-NARX model combined with ridge regression will outperform the four-channel SISO-NARX model in angle estimation accuracy. This was validated in Section 2.6, *Estimation of wrist angle by NARX*. Ridge regression is used to address the issue of unstable and high-variance estimates in the ordinary least squares (OLS) method caused by strong correlations among independent variables. It helps prevent overfitting and improves predictive performance [12]. In this study, the conventional SISO-NARX model was modified to a MISO-NARX model, and the number of channels was increased to eight. As shown in Figure 11, all EMG signals are output, regardless of the direction in which the wrist moves, with multiple channels responding to the same motion. This implies strong correlations among variables. Therefore, ridge regression is expected to enhance the performance of the MISO-NARX model. First, the 4-channel SISO-NARX model was constructed using EMG signals synthesized into a single input, resulting in an RMSE of 32.70 degrees. This was used as a baseline error based on prior research. Next, a four-channel MISO-NARX model was built to compare the performance of single-input and multi-input configurations. The RMSE of the four-channel MISO-NARX model was 29.15 degrees, indicating a slight improvement of 3.55 degrees. However, as shown near the 7 s mark in Figure 16b, the model exhibited significant errors, likely due to reduced system identification accuracy with increased input complexity. Subsequently, an eight-channel MISO-NARX model was analyzed, showing improvement over the four-channel MISO-NARX model. Nevertheless, significant errors were observed in the 0–5 s range in Figure 17. After applying ridge regression to the eight-channel MISO-NARX model, the RMSE improved to 7.80 degrees. These results demonstrate that while the complexity of the eight-channel model introduced additional challenges, ridge regression effectively mitigated overfitting and improved accuracy. This confirms that increasing the number of inputs combined with ridge regression can enhance system performance.

Finally, the practical implementation of the ZPETC+PID controller was evaluated to ensure no issues arise during real-world application. In Hayashi et al.’s study, the delay was reduced from 0.030 s to approximately 0.010 s. In this study, the delay for the servo motor was measured at approximately 0.423 s. Simulations demonstrated that the delay could be reduced by 0.376 s, resulting in a delay of 0.047 s. When applied to the actual servo motor, the delay without the controller was approximately 0.428 s, while the ZPETC+PID controller reduced this to 0.188 s; a reduction of 0.240 s. As mentioned in the introduction, delays exceeding 300 ms can negatively impact user performance in remote operations [6]. With the ZPETC+PID controller implemented, the delay was measured at 0.188 s, further validating the effectiveness of the controller in enhancing responsiveness.

### 4.2. Future Directions

The limitations of the current study are as follows. In this study, EMG and angle data were extracted and used from a single participant. This is because the focus of this research was on validating the effectiveness of the system for controlling a myoelectric prosthesis. In other words, it is assumed that individual differences in EMG are reflected in the NARX model through calibration, based on the idea that prosthetic hands are individual. In future studies, we plan to verify the results when calibrating the same control system with data from other individuals.

The following are three research directions planned for future studies. First, directly controlling the motor using EMG signals, along with verifying the resulting angle and response speed, could further optimize motor responsiveness, allowing for real-time feedback and enhancing user convenience. Second, expanding the system to estimate finger movements would make it possible for the prosthetic hand to perform finer, more precise tasks according to the user’s intent. This would facilitate more natural interactions in daily activities, increasing the practical utility of the prosthesis. Third, it is essential to develop a classifier that can identify which finger is in motion. By identifying the active finger from a single EMG signal and then applying the NARX model, a classifier would enable a more accurate control system and is therefore anticipated to be a critical element in the prosthetic’s effectiveness. These research extensions hold the potential to make prosthetic hands more natural and useful in daily life, significantly enhancing the user experience.

## 5. Conclusions

This study validated its hypotheses by addressing key challenges in myoelectric prostheses. The first hypothesis, that an eight-channel wireless EMG sensor could introduce communication delays while still allowing effective utilization of EMD, was confirmed. Measured delays remained within acceptable limits, demonstrating that EMD can enhance responsiveness even with wireless sensors. The second hypothesis, that the eight-channel MISO-NARX model with ridge regression would outperform the four-channel SISO-NARX model, was also validated. The proposed model significantly improved angle estimation accuracy, reducing RMSE from 32.70 degrees to 7.80 degrees. Furthermore, the implementation of the ZPETC+PID controller effectively minimized response delays, confirming its suitability for real-world application. These findings establish a robust foundation for scalable, real-time prosthetic systems and future expansion to multi-degrees-of-freedom movements.

## Figures and Tables

**Table 1:** The results of applying a 60-degree step input.

Time (s)	Target (°)	Attempt 1	2	3	4	5	6	7	8
0.000	60	0.00	0.00	0.00	0.00	0.00	0.00	0.00	0.00
0.047	60	9.84	10.90	10.20	9.84	10.20	10.28	10.02	9.93
0.094	60	29.88	31.82	30.67	29.88	30.67	30.85	30.50	30.06
0.141	60	49.31	50.89	49.83	49.48	49.66	50.10	49.92	49.48
0.188	60	56.60	57.39	56.69	56.87	55.99	56.60	56.87	56.60
0.235	60	58.01	58.54	57.83	58.10	57.13	57.83	58.01	57.74
0.282	60	58.54	59.06	58.45	58.54	57.66	58.27	58.36	58.10
0.329	60	59.06	59.77	58.97	59.24	58.27	58.89	58.97	58.71
0.376	60	59.59	59.94	59.59	59.68	58.89	59.41	59.50	59.24
0.423	60	59.94	59.94	59.77	59.94	59.06	59.77	59.94	59.68
0.470	60	59.94	59.94	59.77	59.94	59.06	59.85	59.94	59.68
0.517	60	59.94	59.94	59.77	59.94	59.06	59.85	59.94	59.68
0.564	60	59.94	59.94	59.77	59.94	59.06	59.85	59.94	59.68
0.611	60	59.94	59.94	59.77	59.94	59.06	59.85	59.94	59.68

**Table 2:** Coefficients of the plant’s transfer function.

$a_{2}$	$a_{1}$	$a_{0}$	$b_{1}$	$b_{0}$	$c_{1}$
0.1881	−0.7626	1	0.1573	0.2637	$c_{1}$ ^1^

^1^ $c_{1}=b_{0}/b_{1}$.

**Table 3:** Bluetooth latency of the Myo armband.

Average [s]	Minimum [s]	Maximum [s]
0.001078	0.000272	0.034995

**Table 4:** Angle and voltage correlation.

Angle [deg]	Voltage [V]
−90	1.00
−60	1.34
−30	1.68
0	2.01
30	2.34
60	2.68
90	3.02

**Table 5:** RMSE results for ARX model estimation.

Dataset and Test Case	RMSE (deg)
The ARX estimation results for Dataset 1 with an order of 1 are presented below.	21.23
The ARX estimation results for Dataset 2 with an order of 1 are presented below.	13.96
The ARX estimation results for Dataset 1 with an order of 2 are presented below.	18.70
The ARX estimation results for Dataset 2 with an order of 2 are presented below.	12.25

**Table 6:** RMSE results for NARX model with varying intervals *m*.

Dataset	RMSE (deg) m=2	RMSE (deg) m=3	RMSE (deg) m=4	RMSE (deg) m=5
Dataset 1	7.3794	6.0054	5.6440	6.5557
Dataset 2	80.0059	8.9194	8.9021	29.7391
Dataset 3	13.2612	10.2512	13.6647	16.1517
Dataset 4	60.5186	942.3175	20.8698	41.7929

**Table 7:** PID control gains.

$K_{p}$	$K_{i}$	$K_{d}$
0.4837	6.0959	0

## Data Availability

Although the data used in this study were anonymized and cannot identify individual participants, data sharing is not applicable to this article due to privacy concerns.

## Abbreviations

The following abbreviations are used in this manuscript:

EMG	Electromyography
EMD	Electro-Mechanical Delay
NARX	Nonlinear Autoregressive with Exogenous Inputs
ZPETC	Zero Phase Error Tracking Control
RMSE	Root Mean Square Error
PID	Proportional–Integral–Derivative
